# Discrete structural features among interface residue-level classes

**DOI:** 10.1186/1471-2105-16-S18-S8

**Published:** 2015-12-09

**Authors:** Gopichandran Sowmya, Shoba Ranganathan

**Affiliations:** 1 Department of Chemistry and Biomolecular Sciences, Macquarie University, Sydney, NSW 2109 Australia

**Keywords:** Protein-protein interaction (PPI), heterodimers, interface, surface, polarity

## Abstract

**Background:**

Protein-protein interaction (PPI) is essential for molecular functions in biological cells. Investigation on protein interfaces of known complexes is an important step towards deciphering the driving forces of PPIs. Each PPI complex is specific, sensitive and selective to binding. Therefore, we have estimated the relative difference in percentage of polar residues between surface and the interface for each complex in a non-redundant heterodimer dataset of 278 complexes to understand the predominant forces driving binding.

**Results:**

Our analysis showed ~60% of protein complexes with surface polarity greater than interface polarity (designated as class A). However, a considerable number of complexes (~40%) have interface polarity greater than surface polarity, (designated as class B), with a significantly different p-value of 1.66E-45 from class A. Comprehensive analyses of protein complexes show that interface features such as interface area, interface polarity abundance, solvation free energy gain upon interface formation, binding energy and the percentage of interface charged residue abundance distinguish among class A and class B complexes, while electrostatic visualization maps also help differentiate interface classes among complexes.

**Conclusions:**

Class A complexes are classical with abundant non-polar interactions at the interface; however class B complexes have abundant polar interactions at the interface, similar to protein surface characteristics. Five physicochemical interface features analyzed from the protein heterodimer dataset are discriminatory among the interface residue-level classes. These novel observations find application in developing residue-level models for protein-protein binding prediction, protein-protein docking studies and interface inhibitor design as drugs.

## Background

Protein-protein binding is a known phenomenon in complex biological networks. The molecular principle of such binding is often elusive in nature. Understanding its driving forces using known protein complexes is essential. The analysis of existing protein-protein interaction (PPI) complexes from the Protein Data Bank (PDB) [1] is the key to gaining insights into recognition mechanisms and binding principles as reviewed elsewhere [2-6]. Sequence and structural investigations on the existing complexes has been carried out for several decades [3,7-10]. In these extensive surveys, structural features over diverse datasets of protein-protein complexes were typically averaged, obscuring information on individual proteins' structural integrity. Each individual complex is specific and sensitive to binding. Although, non-polar (or hydrophobic) interactions are known to play a major role in contributing to the driving force for binding, in a considerable number of complexes, polar interactions are also observed to contribute abundantly to the formation of a stable interface [11]. Therefore, it is often essential to study the relative difference in surface and interface polarity of each PPI complex to determine the major binding forces at the interface and determine their discriminatory features.

Interfaces are localized regions of surfaces with different physico-chemical properties compared to the rest of the surfaces, thereby driving binding to other molecules. Both physical and chemical features (including hydrophobicity, electrostatic interactions, binding energy, interface size, hydrogen bonds, salt bridges, disulphide bonds, planarity, sphericity, shape complementarity, amino acid chemical groups, and conserved residue clusters) govern the formation of protein interfaces as described elsewhere [7,9,12-18]. The chemical nature of residues forming a protein interface (amino acid residue composition) determines the hydrophobic effect of an interface. Non-polar (or hydrophobic) residues are observed to occur predominantly at the protein interface, playing a major role in contributing to the driving force for binding [7,13]. Interfaces are observed to be less non-polar (or hydrophobic) than the protein interior [13]. The residue composition of protein-protein interfaces was observed to be more similar to the protein surface than the protein interior [9].

Interfaces were observed to be significantly polar as well as non-polar with few charged groups, similar to the characteristics of the protein surface [12]. Structural analysis also revealed that charged and polar amino acids are involved in protein-protein interactions as reviewed elsewhere [19]. Charged and polar residues contributing to binding specificity and complex formation are demonstrated in a number of complexes such as human IL-4, human CD2 and CD58, barnase-barstar, Colicin E9, integrin αvβ6 membrane protein and in intrinsically disordered proteins [20-25]. Shape complementarity, polar interactions, hydrogen bonding and salt bridges are also known to contribute to binding specificity and free energy of binding [17,24,26,27]. Also, charged and aromatic side chains are crucial for binding, determining the cation-pi, electrostatic and aromatic interactions [8]. The role of electrostatics in binding stability of protein-protein complexes is demonstrated [16]. These observations indicate that although PPIs are driven by non-polar interactions at the interface for a majority of complexes, in some cases polar interactions contribute to binding specificity (characteristic of polar residues) and likewise to complex stabilization. Therefore, a study on the relative percentage difference between surface and interface polarities of each protein complex is often essential. In our previous study, we have identified a class of complexes with more polar residues at the interface than the rest of the surface, where binding is mainly polar with a dataset of 198 complexes [11]. This observation has now been extended for an updated yet non-redundant dataset of 278 protein complexes to verify and identify any discriminatory features among these interface residue-level classes.

In this study, we have carried out a comprehensive structural analysis of 278 non-redundant heterodimeric protein complexes from the PDB. We estimated the relative difference in surface and interface polarities of each complex in the dataset, using percentage values of polar residues. This property divides the dataset into two interface classes as also observed in our previous study with a smaller dataset [11]. Class A has less polar residues at the interface than the rest of the surface (~60%) which is the 'classical' definition of a PPI complex and class B has more polar residues at the interface than the rest of the surface (~40%), is 'non-classical.' Therefore, we have investigated essential PPI structural features such as interface area (ΔASA), the relative abundance of polar and non-polar residues at the interface (interface polarity abundance), hydrogen bonds (H-bonds), salt bridges, percentage of charged residues at the interface (interface charged residues%), solvation free energy gain upon interface formation (Δ^i^G), binding energy (BE), and electrostatics among these interface classes and their gleaned features are documented. We identified five key features (ΔASA, interface polarity abundance, interface charged residues%, Δ^i^G and BE) that are significantly different between the interface classes. These novel observations have implications for residue-level characterization of protein complexes to develop models for protein-protein binding prediction and docking studies.

## Methods

### Heterodimer dataset

We created a non-redundant heterodimer dataset of protein complexes from the PDB, using the RCSB PDB's advanced search interface. The following criteria were used for filtering: (i) resolution <= 3Å (ii) protein size >50 residues (iii) contains experimental data (iv) number of chains, entities and oligomeric state is set at 2 (v) devoid of DNA or RNA or a hybrid of such molecules with the protein or otherwise (vi) entries with mutations were not included and (vii) sequence identity cut-off is set to 30%, which is the minimum cut-off available in the PDB. As a second step, the USEARCH program [28] was used to further remove the redundancy among heterodimer complexes at sequence identity cut-off of 20%, as this threshold eliminates remote homology up to 25% sequence identity seen in structures as well [29].

### Interface analysis

The interface of PPI complex is calculated as the change in solvent accessible surface area (ΔASA) upon complex formation. The Surface Racer 5.0 program [30] was used to calculate ASA with a probe radius of 1.4Å and Lee and Richards implementation [31]. Interface residues with ΔASA > 0.1Å^2 ^were considered for this analysis, as defined by Chakrabarti and Janin [32]. ASA was used to determine surface residues of each complex. The amount of polar, non-polar and charged residues at the interface was then estimated for the dataset. The interface polarity abundance (P%-NP%) is measured as the difference in the percentage of polar residues (P%) and percentage of non-polar residues (NP%) at the interface [11].

### Classification based on relative interface-surface polarity

Interfaces are part of protein surface formed upon binding of individual subunits. Each protein complex has a specific composition of polar (P) and nonpolar (NP) residues at the surface (S) and at the interface (I). The distribution of polar and nonpolar residues at the interface of a protein complex describes the nature of the interface and the major driving force for binding. We have calculated the percentage of polar and nonpolar residues at the surface and interface for each complex in the dataset. Polar residues considered in the analysis are R, N, D, E, Q, H, K, S, T, and Y and non-polar residues are A, C, G, I, L, M, F, P, V, and W. Complexes were then grouped based on the relative difference in percentage of polar residues between surface (S) and the interface (I). Complexes with interface polarity less than the surface (represented as S>I) are grouped as class A, and those that have interface polarity greater than the surface (represented as S<I) are grouped as class B [11].

### Intermolecular H-bonds and salt bridges calculation

We calculated the intermolecular hydrogen bonds for the dataset using HBPLUS program [33] at a distance of < 4Å. The H-bonds were extracted such that the donor and acceptor are from two different chains. Salt bridges were calculated using SBION program [34] within a distance of 4Å. The salt bridges were also extracted such that the oppositely charged atoms are from two different chains.

### Δ^i^G and BE calculation

PDBePISA webserver [35] was used to obtain the solvation free energy gain upon interface formation (Δ^i^G, in kcal/mol, with negative Δ^i^G values indicating hydrophobic interface) and for the heterodimer dataset. BE values were calculated using the DCOMPLEX program [36] with the most negative value considered the strongest. The DCOMPLEX program uses DFIRE-based potentials [37] to calculate BE terms, without values for individual components (electrostatic, van der Waals, hydrophobic and entropic terms) contributing to BE. Initially, the program calculates the total atom-atom potential of mean force, G, for each protein structure as follows:

(1) $$G=\frac{1}{2}\underset{i,j}{\sum }\left({ū}_{i,j},r_{i,j}\right)$$

where ū is the atom-atom potential of mean force between two atoms, i and j which are 'r' distance apart. The total is over atomic pairs which are not from the same residue and a K factor is used to avoid double-counting of residue-residue and atom-atom interactions [36]. The binding energy between two interacting proteins A and B can be calculated as follows:

(2) $$\text{Δ}G_{bind}=G_{complex}\left(G_{A}+G_{B}\right)$$

where A and B are considered as two protein structures whose interface residues contribute most to ΔG_bind_. Therefore, DCOMPLEX [36] uses the equation below to calculate BE:

(3) $$\text{Δ}G_{\text{bind}}=\frac{1}{2}\overset{interface}{\underset{ij}{\sum }}\left({ū}_{i,j},r_{i,j}\right)$$

### Electrostatic potential at the interface

The surface electrostatic potential of chain A and chain B of a protein complex was calculated by solving Poisson-Boltzmann equation with dielectric constant (protein) of 4 using DEEPVIEW [38].

### Statistical analysis

The Wilcoxon signed-rank test [39], a non-parametric statistical hypothesis test is used to compare the two interface classes to assess whether the mean ranks for the PPI features in the two classes differ (i.e. it is a paired difference test). The discriminatory PPI features among the two classes were thus tested for statistical significance with p < 0.05 (for the Wilcoxon signed-rank test) in RStudio [40].

### Results and discussion

**Figure 1:**
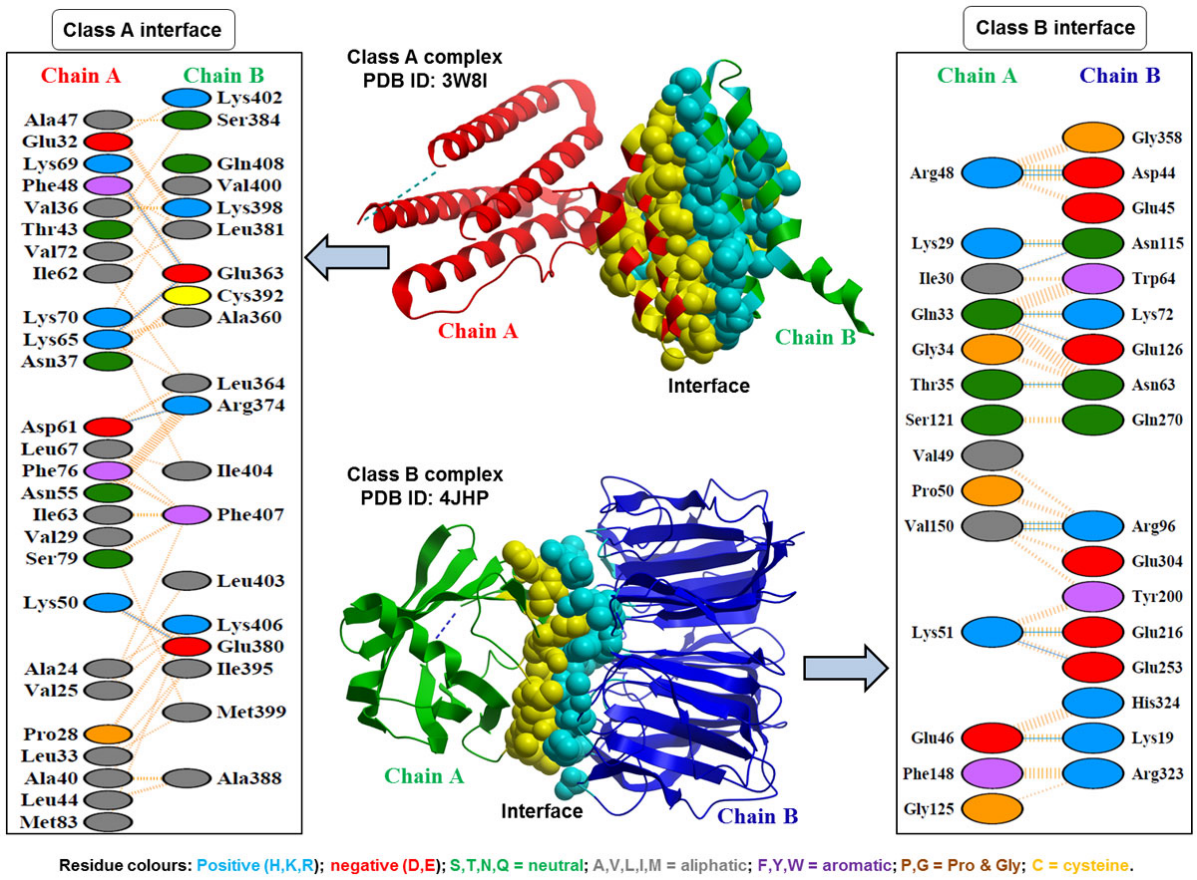
**Examples of PPI interfaces in class A and class B complexes**. The PDBsum [41] interaction analysis represents interaction residues on either chain with residues shown in different colours based on their properties and the coloured lines joining these residues representing the type of interaction between these residues. Class A complex shows a surface polariy of 60.28% and interface polarity of 37.84% (S>I) implying relatively less polar interactions at the interface (or relative abundance of non-polar interactions at the interface). Class B complex shows surface polariy of 50.69% and an interface polarity of 73.21% (S<I) implying relative abundance of polar interactions at the interface (or relatively less non-polar interactions at the interface).

We calculated the amount of polar and non-polar residues at the surface and interface of each protein-protein complex and estimated their relative interface-surface polarities for classification into class A and class B (as described in Materials and Methods section), to determine the type of interactions predominantly driving protein-protein binding. Additional File 1: **Table S1 **shows the heterodimer dataset (278) divided into class A (165) and class B (113). Thus, 59.4% of complexes in our dataset belong to class A (relative surface polarity is greater than interface polarity), where non-polar interactions are predominant at the interface, as previously observed in a number of studies [7,13]. Nevertheless, 40.6% of complexes belong to class B (relative interface polarity is greater than surface polarity), where polar interactions are predominant at the interface, similar to the surface characteristics as also observed [12]. Class A and class B are significantly different with a p-value of 1.66E-45 (using Wilcoxon rank sum test) as shown in Additional File 2: **Figure S1**. Examples of class A and class B complexes representing predominant non-polar and predominant polar interfaces (using the PDBsum [41] interaction analysis) respectively are shown in Figure 1.

### PPI features among class A and class B complexes

**Figure 2:**
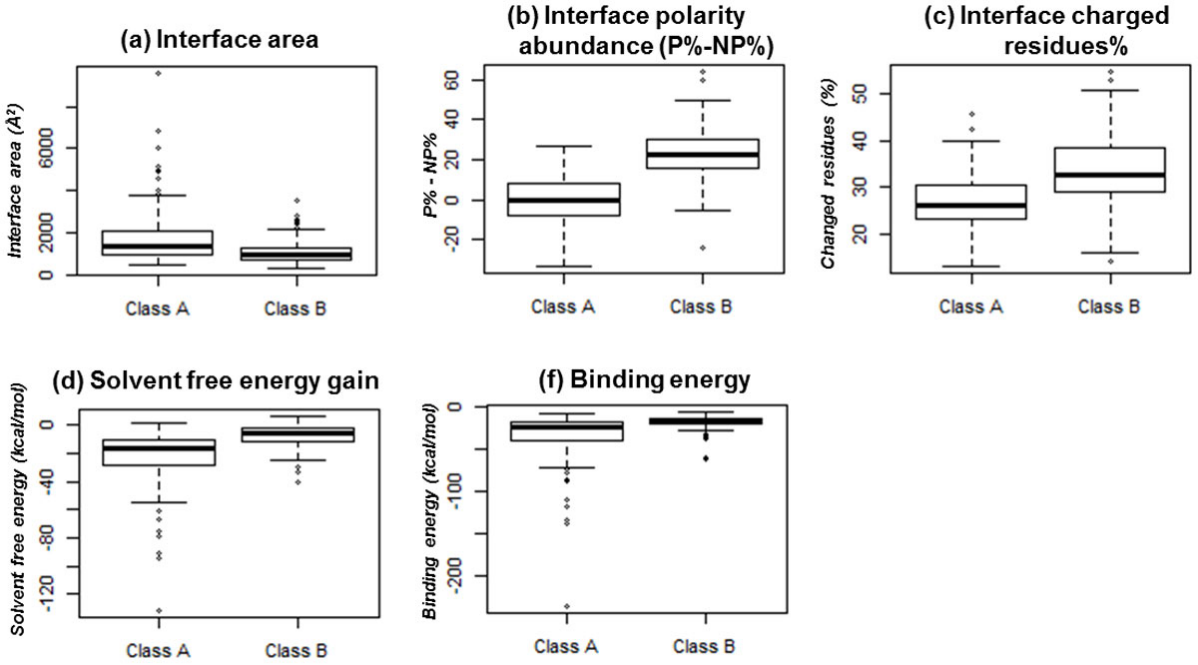
**Distinguishing PPI features among interface classes**. The interface area (ΔASA), interface polarity abundance (P%-NP%), interface charged residues%, solvent free energy gain (Δ^i^G), and BE are shown to distinguish among class A and class B complexes (p-values are shown in Table 1).

We carried out a statistical analysis of all the structural features (described in Materials and Methods section including ΔASA, interface polarity abundance, interface charged residues%, H-bonds, salt bridges, Δ^i^G, BE) in R program (using Wilcoxon rank sum test), to determine whether structural features discriminate among class A and class B complexes. Interestingly, five structural features showed significant difference among the interface classes as shown in Figure 2, with p-value < 0.05 (Table 1). The q-value in Table 1 is the smallest False Discovery Rate (FDR) at which a particular class (class A or class B) would stay on the discriminatory features table. This is not identical to the p-value, which is the smallest false positive rate (FPR) at which a class appears positive on the discriminatory features table. The p-value is much stricter than the q-value. An FDR of 5% (q-value <0.05) is acceptable, which is accepting 5% of erroneous single results, according to Wilcoxon test [39]. These structural features are presented below, along with sets of other correlated properties and electrostatics among classes.

**Table 1:** PPI features distinguishing class A and class B (using Wilcoxon rank sum test).

PPI features	P-value	Q-value
Interface polarity abundance (P%-NP%)	7.01E-30	1.19E-28
Solvent free energy gain (Δ^i^G),	7.43E-18	1.19E-16
Binding energy	2.63E-14	3.68E-13
Interface charged residues%	6.58E-13	8.55E-12
Interface area	1.31E-08	1.57E-07

### Interface polarity abundance among classes

Protein interfaces are composed of both polar and non-polar residues. Some interfaces are abundant in non-polar residues while few others are abundant in polar residues. The interface polarity abundance (P%-NP%) measure is significantly different among the interface classes with p = 7.01E-30 (Table 1 and Figure 2).

### Δ^i^G among classes

The solvation free energy gain upon interface formation (Δ^i^G) is a measure of the interface stability in protein complexes [35]. The Δ^i^G values are significantly different among interface classes with p = 7.43E-18 (Table 1) as shown in Figure 2.

### BE among classes

The strength of binding among class A and class B complexes is estimated as a measure of BE in kcal/mol. The BE values are relatively stronger for class A complexes (average BE is -33.99 kcal/mol), as compared to class B complexes (average BE is -17.94 kcal/mol). The BE values are significantly different among interface classes with p = 2.63E-14 (Table 1) as shown in Figure 2.

### Interface charged residues among classes

The percentage of charged residues at the interface is estimated for both classes. The interface charged residues% is significantly different among interface classes with p = 6.58E-13 (Table 1) as shown in Figure 2.

### ΔASA among classes

The interface area (ΔASA) of a complex is an important structural characteristic of PPI. We observed that class A complexes demonstrate comparatively larger interfaces than class B complexes. The ΔASA is significantly different among the classes with p = 1.31E-08 (Table 1 and Figure 2).

### Other correlations of interface features among classes

The stability of protein-protein binding depends on the number of hydrogen bonds and salt bridges formed between the two interacting subunits. Class A complexes show high correlation between intermolecular H-bonds and interface area (r = 0.9) as previously observed [7,42]. However, class B complexes alone show reduced trends (r = 0.73) between intermolecular H-bonds and interface area (Additional File 3: **Figure S2)**, indicating that low quality of intermolecular hydrogen bonds is a characteristic of the large number of polar or charged residues across protein interfaces as previously observed [17]. Although salt bridges showed no distinguishing trends among classes, we observed that class B complexes are rich in salt bridges (average number of salt bridges is 6.5), as compared to class A complexes (average number of salt bridges is 5.8).

The BE values are proportional to interface area in the dataset (r = 0.96, shown in Additional File 4: **Figure S3**) as previously observed [43]. The Δ^i^G values show relatively less correlation with interface area in class B complexes (r = -0.62) as compared to class A complexes (r = -0.92, Additional File 5: **Figure S4**). Moreover, the Δ^i^G and BE is highly correlated among the dataset (r = 0.88) and class A (r = 0.91), however shows limited correlation among class B (r = 0.55, Additional File 6: **Figure S5**).

### Electrostatic visualization maps among protein interface classes

**Figure 3:**
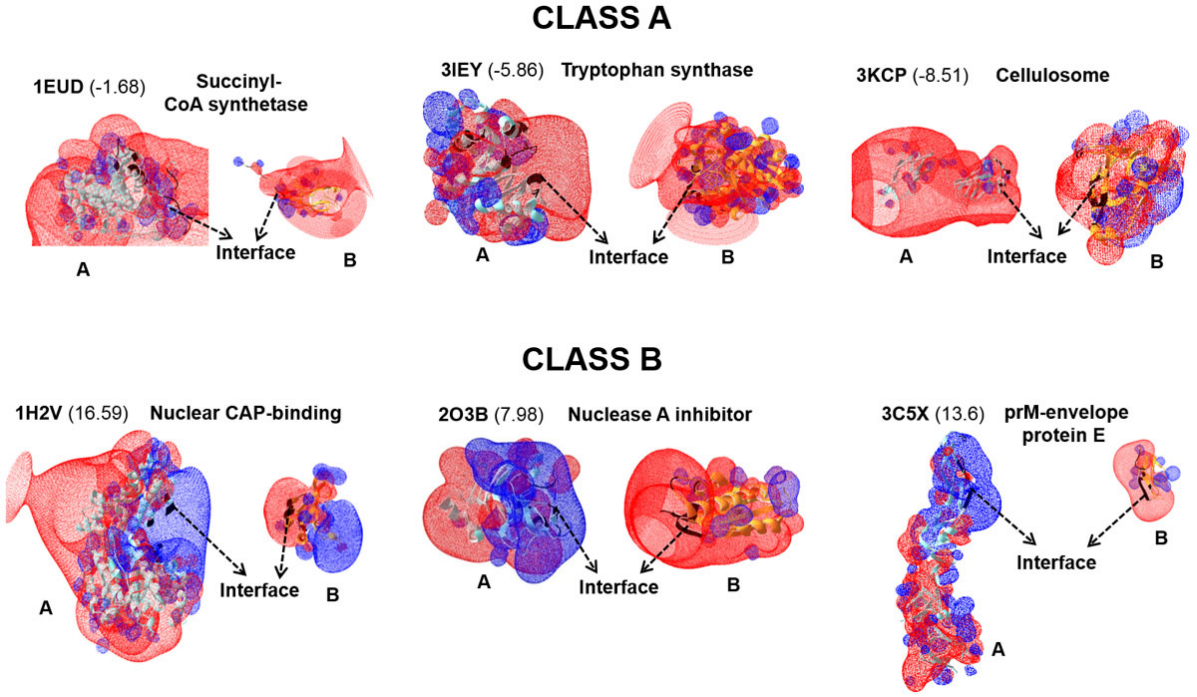
**Surface electrostatics distribution of Class A and Class B complexes using DEEPVIEW**. The heterodimer complexes are shown in cartoon representation with chain A in cyan, chain B in orange and interface residues colored in black. PDB IDs and protein names are given for each complex along with I-S values (numbers in parenthesis represent (P%-NP%), the interface polarity abundance). The electrostatic potential images of class A complexes show that the interface of chain A and of chain B have same charges (similar colors), suggesting electrostatic energy may not favor protein binding in class A complexes. The electrostatic potential images of class B complexes show that the interface of chain A and chain B have opposite charges (different colors); suggesting electrostatic energy favors protein binding in class B complexes.

We have studied the surface electrostatic potential solving Poisson-Boltzmann equation using DEEPVIEW for a few examples of class A and class B complexes. This shows common surface electrostatics at work amongst the class A and amongst the class B complexes. Interestingly, the class A complexes demonstrate similar distribution of charges at the protein interfaces of both chains, suggesting electrostatic energy may not contribute to binding energy among class A complexes. However, class B complexes show opposite charge distributions at the protein interfaces, suggesting electrostatic energy plays an important role in PPIs among class B complexes as shown in Figure 3. Therefore, the surface electrostatic potential maps give quick visual clues for identifying or distinguishing class A and class B complexes.

## Conclusions

Structural analyses of known protein interfaces help in understanding the molecular principals of PPIs. Therefore, a comprehensive analysis of known structural interfaces of 278 complexes was carried out and their gleaned features are documented in this study. It is realized that each complex type is unique, specific and sensitive to binding. Nonetheless, there is a considerable degree of observed pattern among protein interface classes. We report two classes of interfaces, one class with less polar residues and the other class with more polar residues compared to the surfaces in bound state. The surfaces of proteins are quite polar and therefore, it is perhaps not surprising that some interfaces are polar as well and that PPI complex forms due to interactions among charged and polar residues. Thus, the need for a residue-level characterization of the interface is crucial in addition to other structural features. We document five discriminatory features (interface area, interface polarity abundance (P%-NP%), interface charged residues%, solvent free energy gain upon interface formation (Δ^i^G), and binding energy) among the interface residue-level classes. This is a first attempt towards classifying the complexes based on interface residue-level classes for the characterization of PPI features amongst these classes. These observations corroborate the need for classification of complexes in determining their combinatorial features and drawing consensus for common patterns in protein-protein recognition. These results provide molecular insights for protein-protein binding towards the development of residue-level prediction models in future studies. Additionally, mutation experiments using hot spot residue databases [44] and detailed interface residue characterization (cation-pi, electrostatic and aromatic interactions [8]) will further strengthen this study, for individual structures. Furthermore, extending this analysis for a larger dataset with a combined formulation of atomic and residue level features in future studies may improve protein-protein docking.

## Abbreviations

PPI, Protein-Protein Interaction; Δ^i^G, solvent free energy gain upon interface formation; BE, Binding Energy; H-bonds, Hydrogen bonds; PDB, Protein Data Bank; ΔASA, interface area; P, Polar residues; NP, Non-polar residues; S, Surface polarity; I, Interface Polarity.

## Competing interests

The authors declare that they have no competing interests.

## Authors' contributions

Conceived and designed the experiment: GS. Data collected, analyzed and drafted the manuscript: GS. Interpretation of results and finalizing the manuscript: GS and SR. All authors read and approved the final manuscript.

## Supplementary Material

Additional file 1 Table S1: Heterodimer dataset (278) divided into interface classes based on residue level relative surface-interface polarity. The PDB code is shown along with the specific chains used in this study. Click here for file

Additional file 2 Figure S1: Class A and Class B are significantly different. The boxplot depicts class A and class B significantly different with a p-value of 1.66E-45 (using Wilcoxon rank sum test). Click here for file

Additional file 3 Figure S2: Intermolecular H-bonds shows relatively low correlation with interface area in class B. Hydrogen bonds at the protein interface are highly correlated to interface area in the dataset (r = 0.88) and class A (r = 0.9), however shows relatively lower trends (r = 0.73) in class B. Click here for file

Additional file 4 Figure S3: Binding energy is highly correlated to interface area. BEs at the protein interfaces are highly correlated to interface area with r = -0.96. Click here for file

Additional file 5 Figure S4: Solvation free energy gain upon interface formation (Δ^i^G) shows limited correlation with interface area in class B complexes. Δ^i^G shows high correlation with interface area in (a) heterodimer dataset (r = -0.88), and (b) class A (r = -0.92), however shows limited correlation in (c) class B complexes (r = -0.62). Click here for file

Additional file 6 Figure S5: BE shows limited correlated with Δ^i^G in class B. Binding energies at the protein interfaces are highly correlated to solvation free energy gain upon interface formation (Δ^i^G) in the dataset (r = 0.88) and class A (r = 0.91), however shows limited correlation between BE and Δ^i^G in class B (r = 0.55). Click here for file

## Declaration

Publication charges for this article have been funded by Macquarie University.

This article has been published as part of *BMC Bioinformatics *Volume 16 Supplement 18, 2015: Joint 26th Genome Informatics Workshop and 14th International Conference on Bioinformatics: Bioinformatics. The full contents of the supplement are available online at http://www.biomedcentral.com/bmcbioinformatics/supplements/16/S18.
