# Supplementary material for: Discrete structural features among interface residue-level classes
Source: BMC Bioinformatics. 2015 Dec 9;16(Suppl 18):S8. doi: 10.1186/1471-2105-16-S18-S8 (PMC4682381; doi:10.1186/1471-2105-16-S18-S8)
Supplement: Additional file 1 — Table S1: Heterodimer dataset (278) divided into interface classes based on residue level relative surface-interface polarity. The PDB code is shown along with the specific chains used in this study. [file 1471-2105-16-S18-S8-S1.pdf]

Additional file 1

Discrete structural features among interface residue-level classes

Gopichandran Sowmya, Shoba Ranganathan

Table S1: Heterodimer dataset (278) divided into interface classes based on residue level relative surface-interface polarity. The PDB code is shown along with the specific chains used in this study.

Class A (165) [S>I]						
1E44 [A, B]	1YKH [A, B]	2WD5 [A, B]	3EGV [A, B]	3ML1 [A, B]	4AWX [A, B]	4HPL [A, B]
1EUD [A, B]	1Z0J [A, B]	2XFG [A, B]	3EP6 [B, A]	3MXN [A, B]	4B8A [A, B]	4HST [A, B]
1FS0 [E, G]	1Z5Y [D, E]	2ZFD [A, B]	3F62 [A, B]	3NW0 [B, A]	4BJJ [A, B]	4HT3 [A, B]
1GK9 [A, B]	2CG5 [A, B]	2ZIV [A, B]	3FGR [A, B]	3NYB [A, B]	4BMP [A, B]	4I1S [A, B]
1H32 [A, B]	2D74 [A, B]	2ZSI [A, B]	3FPU [A, B]	3OJA [A, B]	4C9B [A, B]	4JE3 [A, B]
1JEQ [A, B]	2DYO [A, B]	3A2F [A, B]	3GA9 [L, S]	3OSS [C, D]	4CBU [A, G]	4JEH [A, B]
1JKG [A, B]	2F4M [A, B]	3A8G [A, B]	3H7H [A, B]	3PGE [A, B]	4CGY [A, B]	4KHA [A, B]
1JMA [B, A]	2FH5 [A, B]	3AA7 [A, B]	3HZH [A, B]	3PV6 [A, B]	4CT0 [A, B]	4KMO [A, B]
1LSH [A, B]	2FTX [A, B]	3ABE [C, Z]	3IEY [A, B]	3Q87 [A, B]	4CXF [A, B]	4L2I [A, B]
1N1J [A, B]	2G2S [A, B]	3AON [A, B]	3IF8 [A, B]	3QN1 [A, B]	4DBG [A, B]	4M69 [A, B]
1NME [A, B]	2GA9 [A, D]	3AQF [A, B]	3JTQ [A, B]	3R07 [A, C]	4DEY [B, A]	4M6W [A, B]
1NRJ [A, B]	2GSK [A, B]	3AU4 [A, B]	3K1R [A, B]	3R24 [A, B]	4E4W [A, B]	4MRT [C, A]
1OF5 [A, B]	2H9A [A, B]	3AYH [A, B]	3K8P [C, D]	3REQ [A, B]	4EGC [A, B]	4NFU [A, B]
1OO0 [A, B]	2OMZ [A, B]	3B0C [T, W]	3KCP [A, B]	3RGW [L, S]	4ETP [A, B]	4NQW [A, B]
1ORY [A, B]	2OZN [A, B]	3B0Z [A, B]	3KF6 [A, B]	3SHG [A, B]	4EUK [A, B]	4O8Y [A, B]
1R0R [E, I]	2P1M [A, B]	3BS5 [A, B]	3KXC [A, C]	3T5X [A, B]	4EYY [R, Q]	
1R8O [A, B]	2PA8 [D, L]	3BTP [A, B]	3L91 [A, B]	3TBI [A, B]	4F6U [A, B]	
1UGH [E, I]	2PQN [A, B]	3CKI [A, B]	3LBX [A, B]	3VYR [A, B]	4F9C [A, B]	
1US7 [A, B]	2QSF [A, X]	3CPT [A, B]	3LF4 [A, B]	3VZ9 [B, D]	4G1M [A, B]	
1V74 [A, B]	2QWO [A, B]	3CQC [A, B]	3LQC [A, B]	3W8I [A, B]	4G6T [A, B]	
1VRA [A, B]	2RAW [A, B]	3CX8 [A, B]	3M1C [A, B]	3ZET [A, B]	4G94 [A, B]	
1WPX [A, B]	2V3B [A, B]	3DBO [A, B]	3M7F [A, B]	3ZVQ [A, B]	4GDX [A, B]	
1WQJ [B, I]	2V6X [A, B]	3DGP [A, B]	3MCB [A, B]	4A5U [A, B]	4GVB [A, B]	
1XEW [X, Y]	2VN6 [A, B]	3DPL [C, R]	3MJ7 [A, B]	4AP2 [A, B]	4H4K [A, C]	
1XOU [A, B]	2VSM [A, B]	3DRA [A, B]	3MKR [A, B]	4AT7 [A, B]	4HNX [A, B]	

**Class B (113) [S<I]**						
1A22 [A, B]	1ZBX [A, B]	2QKL [A, B]	3EI3 [A, B]	3NV0 [A, B]	3VRD [A, B]	4GQ2 [M, P]
1ARO [P, L]	1ZHH [A, B]	2V8S [E, V]	3F6Q [A, B]	3NVN [A, B]	3VU9 [A, B]	4HFF [A, B]
1AY7 [A, B]	2APO [A, B]	2VDB [A, B]	3FJU [A, B]	3NY7 [A, B]	3W9C [A, B]	4IU2 [A, B]
1DJ7 [A, B]	2B42 [A, B]	2VLQ [A, B]	3FMO [A, B]	3O2P [A, E]	3WA5 [A, B]	4IYP [A, C]
1GL4 [A, B]	2BLF [A, B]	2Z5B [A, B]	3FPN [A, B]	3O3O [A, B]	3ZNZ [A, B]	4J38 [A, B]
1H2V [C, Z]	2CKL [A, B]	2Z64 [A, C]	3FQD [A, B]	3OG6 [A, B]	3ZYI [A, B]	4JHP [B, C]
1KA9 [H, F]	2FCW [A, B]	3A4U [A, B]	3GB8 [A, B]	3OJM [A, B]	4BI8 [A, B]	4K12 [A, B]
1M1E [A, B]	2FHZ [A, B]	3ANW [A, B]	3GC3 [A, B]	3ONA [A, B]	4BL7 [A, B]	4KBM [A, B]
1NPE [A, B]	2FOM [A, B]	3AWU [A, B]	3HHM [A, B]	3OQ3 [A, B]	4C2A [A, B]	4KT1 [A, B]
1SVD [A, M]	2HDI [A, B]	3AXJ [A, B]	3KLD [A, B]	3OUN [A, B]	4DRI [A, B]	4KT3 [A, B]
1T0P [A, B]	2HRK [A, B]	3BEG [A, B]	3KYJ [A, B]	3QQ8 [A, B]	4DVG [A, B]	4LV5 [A, B]
1T6B [X, Y]	2IW5 [A, B]	3C5X [A, C]	3MCA [A, B]	3RNQ [B, A]	4EMJ [A, B]	
1USU [A, B]	2O2V [A, B]	3CLS [C, D]	3MP7 [A, B]	3SBT [A, B]	4F48 [A, B]	
1WMH [A, B]	2O3B [A, B]	3D3B [A, J]	3MWD [A, B]	3THO [A, B]	4FZV [A, B]	
1XG2 [A, B]	2P45 [A, B]	3DI3 [A, B]	3N1M [B, C]	3TU3 [A, B]	4G7X [A, B]	
1Z3E [A, B]	2PTT [A, B]	3DLQ [I, R]	3N40 [P, F]	3V8X [A, B]	4GAF [A, B]	
1Z92 [A, B]	2QC1 [A, B]	3DSS [A, B]	3N4I [A, B]	3VF0 [A, B]	4GED [A, B]	

S – Surface polarity; I – Interface polarity
