# Supplementary material for: Discrete structural features among interface residue-level classes
Source: BMC Bioinformatics. 2015 Dec 9;16(Suppl 18):S8. doi: 10.1186/1471-2105-16-S18-S8 (PMC4682381; doi:10.1186/1471-2105-16-S18-S8)
Supplement: Additional file 2 — Figure S1: Class A and Class B are significantly different. The boxplot depicts class A and class B significantly different with a p-value of 1.66E-45 (using Wilcoxon rank sum test). [file 1471-2105-16-S18-S8-S2.pdf]

Additional file 2

Discrete structural features among interface residue-level classes

Gopichandran Sowmya, Shoba Ranganathan

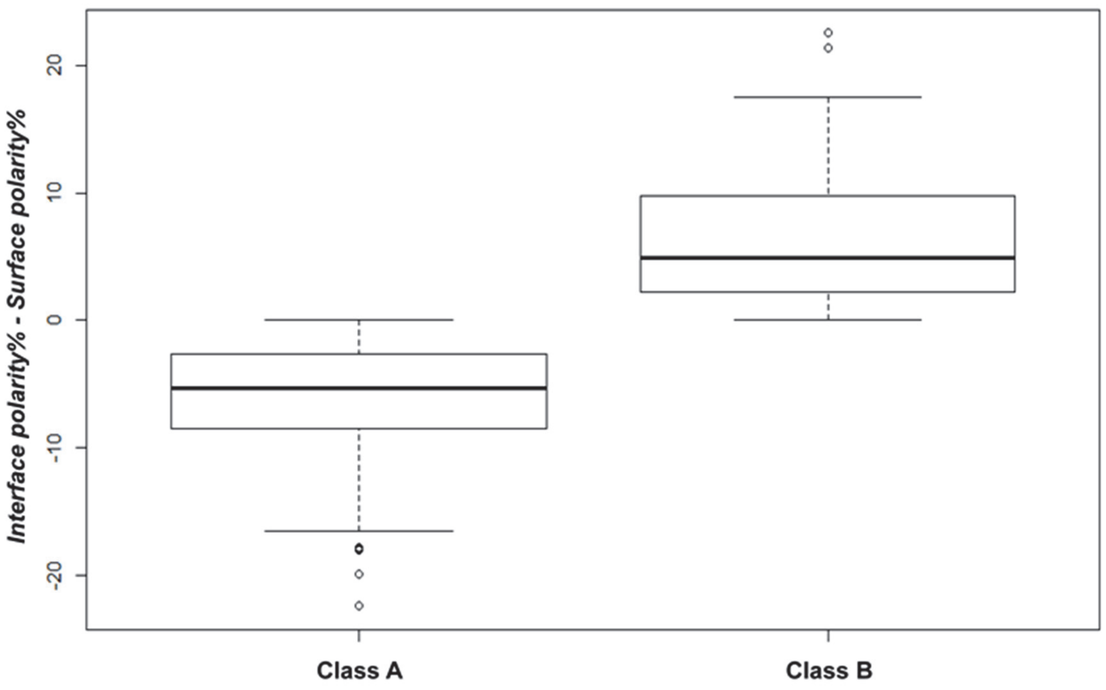

Figure S1: Class A and Class B are significantly different. The boxplot depicts class A and class B significantly different with a p-value of 1.66E-45 (using Wilcoxon rank sum test).
