# Supplementary material for: Discrete structural features among interface residue-level classes
Source: BMC Bioinformatics. 2015 Dec 9;16(Suppl 18):S8. doi: 10.1186/1471-2105-16-S18-S8 (PMC4682381; doi:10.1186/1471-2105-16-S18-S8)
Supplement: Additional file 3 — Figure S2: Intermolecular H-bonds shows relatively low correlation with interface area in class B. Hydrogen bonds at the protein interface are highly correlated to interface area in the dataset (r = 0.88) and class A (r = 0.9), however shows relatively lower trends (r = 0.73) in class B. [file 1471-2105-16-S18-S8-S3.pdf]

Additional file 3

Discrete structural features among interface residue-level classes

Gopichandran Sowmya, Shoba Ranganathan

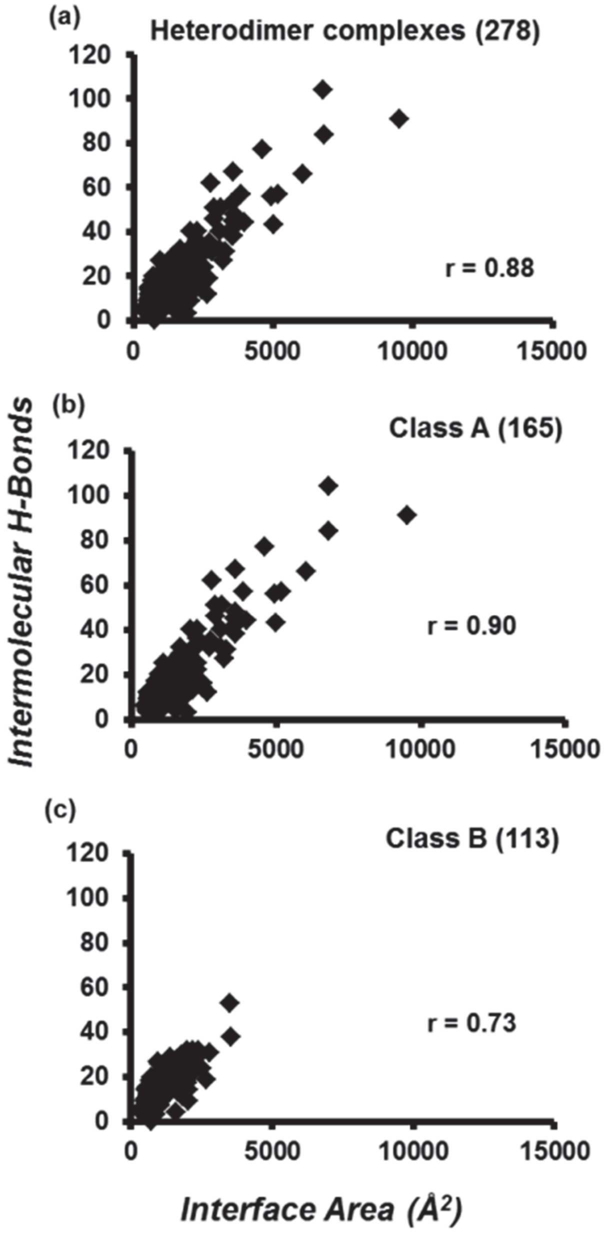

Figure S2: Intermolecular H-bonds shows relatively low correlation with interface area in class B. Hydrogen bonds at the protein interface are highly correlated to interface area in the dataset (r = 0.88) and class A (r = 0.9), however shows relatively lower trends (r = 0.73) in class B.
