# Supplementary material for: Discrete structural features among interface residue-level classes
Source: BMC Bioinformatics. 2015 Dec 9;16(Suppl 18):S8. doi: 10.1186/1471-2105-16-S18-S8 (PMC4682381; doi:10.1186/1471-2105-16-S18-S8)
Supplement: Additional file 4 — Figure S3: Binding energy is highly correlated to interface area. BEs at the protein interfaces are highly correlated to interface area with r = -0.96. [file 1471-2105-16-S18-S8-S4.pdf]

# Additional file 4

# Discrete structural features among interface residue-level classes

**Gopichandran Sowmya, Shoba Ranganathan**

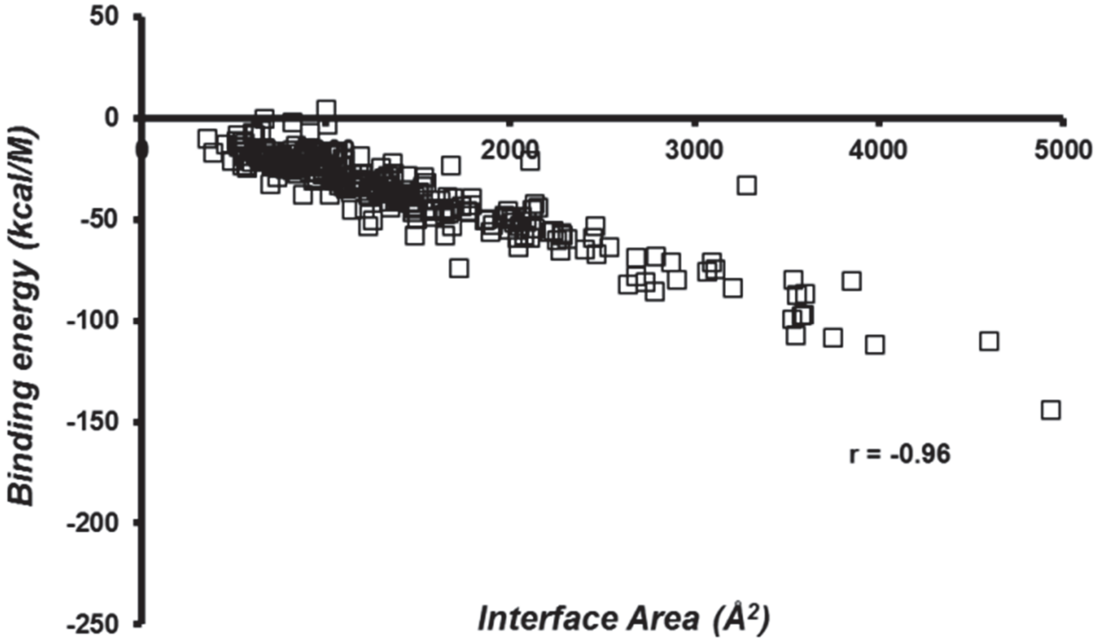


**Figure S3: Binding energy is highly correlated to interface area.** BEs at the protein interfaces are highly correlated to interface area with r = -0.96.
