# Supplementary material for: Discrete structural features among interface residue-level classes
Source: BMC Bioinformatics. 2015 Dec 9;16(Suppl 18):S8. doi: 10.1186/1471-2105-16-S18-S8 (PMC4682381; doi:10.1186/1471-2105-16-S18-S8)
Supplement: Additional file 5 — Figure S4: Solvation free energy gain upon interface formation (ΔiG) shows limited correlation with interface area in class B complexes. ΔiG shows high correlation with interface area in (a) heterodimer dataset (r = -0.88), and (b) class A (r = -0.92), however shows limited correlation in (c) class B complexes (r = -0.62). [file 1471-2105-16-S18-S8-S5.pdf]

Additional file 5

Discrete structural features among interface residue-level classes

Gopichandran Sowmya, Shoba Ranganathan

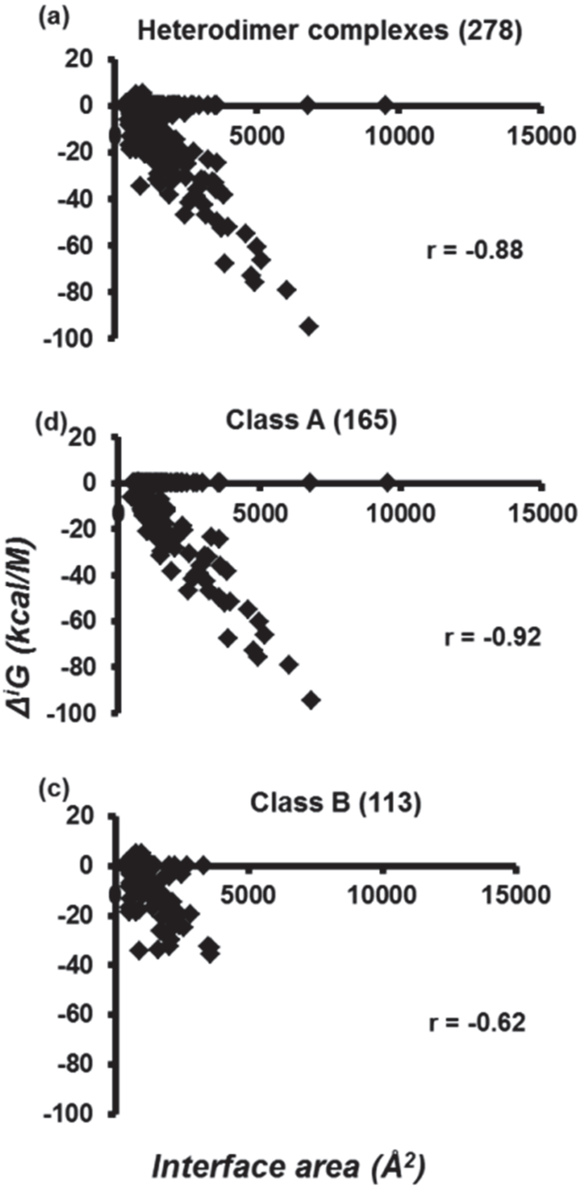

Figure S4: Solvation free energy gain upon interface formation ($\Delta^i G$) shows limited correlation with interface area in class B complexes. $\Delta^i G$ shows high correlation with interface area in (a) heterodimer dataset (r = -0.88), and (b) class A (r = -0.92), however shows limited correlation in (c) class B complexes (r = -0.62).
