# Supplementary material for: Discrete structural features among interface residue-level classes
Source: BMC Bioinformatics. 2015 Dec 9;16(Suppl 18):S8. doi: 10.1186/1471-2105-16-S18-S8 (PMC4682381; doi:10.1186/1471-2105-16-S18-S8)
Supplement: Additional file 6 — Figure S5: BE shows limited correlated with ΔiG in class B. Binding energies at the protein interfaces are highly correlated to solvation free energy gain upon interface formation (ΔiG) in the dataset (r = 0.88) and class A (r = 0.91), however shows limited correlation between BE and ΔiG in class B (r = 0.55). [file 1471-2105-16-S18-S8-S6.pdf]

Additional file 6

Discrete structural features among interface residue-level classes

Gopichandran Sowmya, Shoba Ranganathan

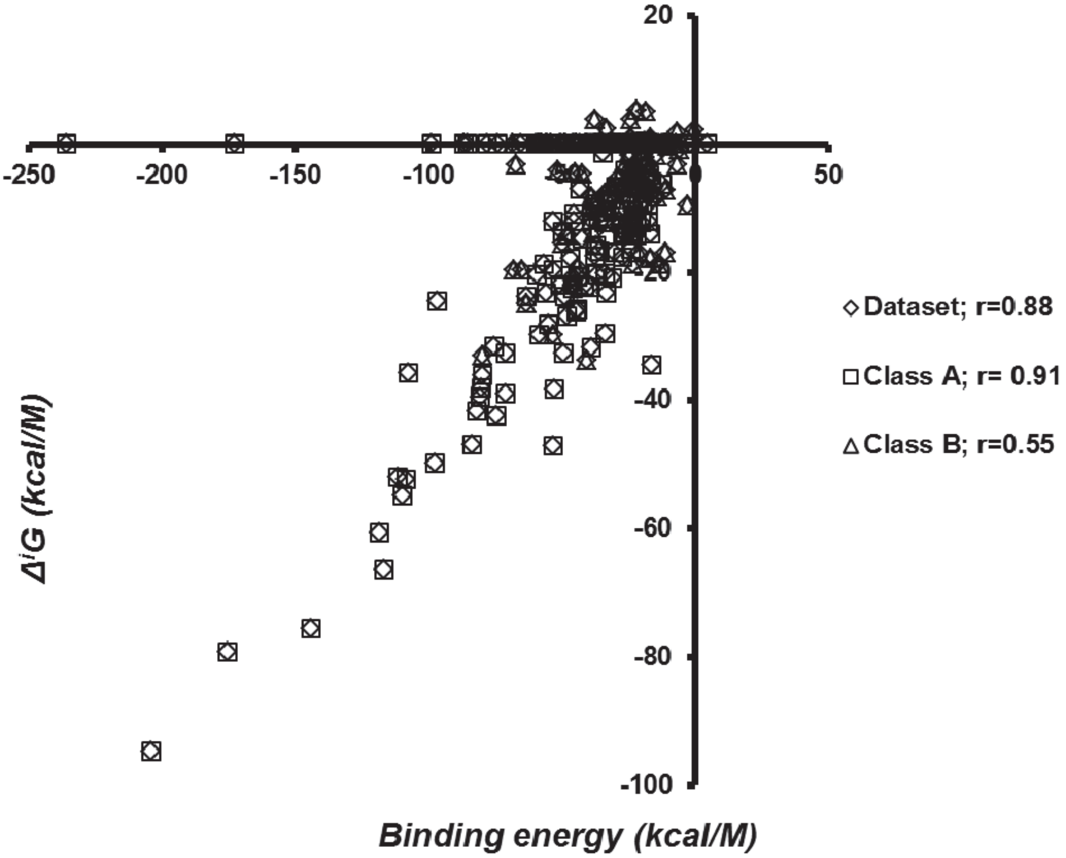

Figure S5: BE shows limited correlated with $\Delta^{i}G$ in class B. Binding energies at the protein interfaces are highly correlated to solvation free energy gain upon interface formation ($\Delta^{i}G$) in the dataset (r = 0.88) and class A (r = 0.91), however shows limited correlation between BE and $\Delta^{i}G$ in class B (r = 0.55).
